# Short-Term Effects of Carbonaceous Components in PM_2.5_ on Pulmonary Function: A Panel Study of 37 Chinese Healthy Adults

**DOI:** 10.3390/ijerph16132259

**Published:** 2019-06-26

**Authors:** Shichun Huang, Huan Feng, Shanshan Zuo, Jingling Liao, Mingquan He, Masayuki Shima, Kenji Tamura, Yang Li, Lu Ma

**Affiliations:** 1 Department of Epidemiology and Health Statistics, School of Health Sciences, Wuhan University, Wuhan 430071, China; 2 Department of Public Health, Wuhan University of Science and Technology School of Medicine, Wuhan 430081, China; 3 Department of Public Health, Hyogo College of Medicine, Nishinomiya, Hyogo 663-8501, Japan; 4 Environmental Health Sciences Division and Integrated Health Risk Assessment Section, National Institute for Environmental Studies, Tsukuba, Ibaraki 305-8506, Japan; 5 Hubei Provincial Center for Diseases Prevention and Control, Wuhan 430079, China

**Keywords:** carbonaceous components, fine particulate matter, healthy adults, respiratory function, mixed-effect model

## Abstract

Objectives: To explore the health effects of indoor/outdoor carbonaceous compositions in PM_2.5_ on pulmonary function among healthy students living in the local university campus. Methods: Daily peak expiratory flow (PEF) and forced expiratory volume in 1 second (FEV_1_) were measured among 37 healthy students in the morning and evening for four two-week periods. Concurrent concentrations of indoor and outdoor PM_2.5_ (particulate matter with an aerodynamic diameter ≤ 2.5μm), carbonaceous components in PM_2.5_, ambient temperature, and relative humidity in the study area were also obtained. Mixed-effects model was applied to evaluate the associations between carbonaceous components and lung function. Different lags for the carbonaceous components were investigated. Results: In single-pollutant model, a 10 μg/m^3^ increase of indoor and outdoor EC (elemental carbon) associated with −3.93 (95%*CI*: −6.89, −0.97) L/min and −3.21 (95%*CI*: −5.67, −0.75) L/min change in evening PEF at lag 0 day, respectively. Also, a 10 μg/m^3^ increase of indoor and outdoor POC (primary organic carbon) concentration was significantly associated with −5.82 (95%*CI*: −10.82, −0.81) L/min and −7.32 (95%*CI*: −12.93, −1.71) L/min change of evening PEF at lag 0 day. After adjusting total mass of PM_2.5_, indoor EC consistently had a significant adverse impact on evening PEF and FEV_1_ at lag3 day and a cumulative effect at lag0-3 day. Conclusions: This study suggests that carbonaceous components in PM_2.5_ indeed have impacts on pulmonary function among healthy young adults especially on evening PEF. Thus, the local mitigation strategies on pollution are needed.

## 1. Introduction

Air pollution and particulate matter (PM) are widely known for their deleterious effects on human health. As previously reported, they increased the risks of cardiopulmonary disease [1,2], the risks of depression in general population [3] and loss of life expectancy [4], and increased mortality burden [5]. Lung function is a noninvasive and reliable measure of respiratory health [6]. Many studies have investigated the associations between PM and lung function. In healthy primary school and non-smoking healthy adults, short-term exposure to PM was negatively associated with FEV_1_ and FEV_1_/FVC ratio [7,8]. Even at relatively low levels of PM pollution, decreased lung function was observed [9]. Although the risk of PM air pollution to each individual is small, given the high prevalence of exposure, it may have severe impact on public health [10]. However, PM is a complex mixture of solid and liquid particles of various sizes and compositions [10], and it is difficult to determine which constituents dominate the effects of PM on lung function [11]. 

Evidenced with previous epidemiological studies, carbonaceous contents in PM_2.5_ posed risks to lung function in adults with asthma [12] and aging population with or without chronic obstructive pulmonary disease (COPD) [11,13,14]. Carbonaceous aerosols generally contained organic carbon (OC) and elemental carbon (EC) [15]. EC, a similar measure of black carbon (BC) [16], is usually coated by nitroso compounds and particle-bound PAHs (polycyclic aromatic hydrocarbons) which are evidenced carcinogenic to animals [15,17]. OC, composition of primary organic carbon (POC) and secondary organic carbon (SOC), also includes PAHs and other possible mutagenic and carcinogenic components [17]. In COPD elders, EC was strongly associated with reduced FEV_1_ and PEF [11], while OC was inversely associated with PEF or FEV1 in specific regression models [11]. Personal exposure of EC-OC was inversely associated with morning FEV_1_ in children with asthma [16]. In subjects with respiratory disease who were also vulnerable, no significant associations were found between BC and changes in spirometry either [18]. Nevertheless, these studies were mostly performed among vulnerable population; attention given to investigate potential health impacts from SOA (secondary organic aerosol) exposure was limited, and health effects attributable to organic carbon components were not well understood.

People worldwide spend most of their time indoors, and this is an important environment for exposure to air pollutants such as PM. Indoor pollutants are transported from outdoors via ventilation, infiltration [19], and soil adhering to clothes and then releasing into indoor air [20]. It has been documented that indoor BC of outdoor origin leads to oxidative burdens on COPD patients [21]. In healthy adults, increased indoor fine particles from certain sources may be associated with small decrements of FEV_1_ and MEF_25%-75%_ [22]. However, previous indoor and ambient air research tended to be conducted in isolation [23]. Nondifferential measurement error was inevitable when using outdoor air pollution as surrogate of indoors. Therefore, indoor air quality should be considered when estimating health effects of air pollution.

This study aimed to elucidate the health effects of indoor/outdoor carbonaceous compositions in PM_2.5_ on pulmonary function among healthy young adults. 

## 2. Materials and Methods

### 2.1. Study Participants and Design

A panel of 37 healthy students (18 male, 19 female) aged from 19 to 21 were recruited from Wuhan University School of Medicine [24]. These students were in the same major and were cluster sampled to enroll in the panel study. They were nonsmokers, without cardiopulmonary and other chronic diseases. Individual information including age, gender, height, and weight were collected. 

18 male students lived in two adjacent dormitories on fifth floor, while 19 female students lived in another two adjacent dormitories of the second floor. The layouts and indoor environments of all rooms were similar. There were no kitchens in dormitories and cooking was not allowed. Participants’ daily routines were simple and they spent most of time in campus, and their air pollution exposure was similar. Besides, they were asked to not leave the campus when possible during the study period for the purpose of homogeneity of exposure among them. We performed the study in four periods: in autumn (29 October 2009 – 11 November 2009), in winter (23 December 2009 – 5 January 2010), in spring (24 March 2010 – 6 April 2010), and in summer (24 July 2010 –6 August 2010). Each period lasted for 14 days and all subjects participated in each of the four periods. This study was approved by the Ethics Review Board of Wuhan University, and written informed consent was obtained from all participants before the study began. 

### 2.2. Measurement

#### 2.2.1. Lung Function Measurement

All of subjects were well-trained by professional technicians for self-administered spirometry test before the study. All participants performed spirometry tests at 7:30 a.m. and 10:00 p.m. every day during each period using the electronic PEF and FEV_1_ diary meter (Model 2110, Vitalograph Ltd., Buckingham, UK). Lung function was measured according to the European Respiratory Society (ERS) guidelines [25]. Individually, at least five lung function measurements were taken each time to ensure at least two that were within 10% of each other. Measurements were performed in standing upright position. Participants were required to contact the mouthpiece with their lips tightly, with a pinched nose and to exhale as hard and fast as possible. Every instrument was calibrated before the spirometry test. To ensure the quality of data, there was a certain technician for each room to supervise the PEF and FEV_1_ data collection and recordation.

#### 2.2.2. Environmental Measurement

The distance between these dormitories and main road were approximately 200 meters. East Lake located on the east side of dormitories, within a distance of about 500 meters. We selected one male dormitory and one female dormitory, and settled two mini pumps indoor and outdoor respectively in both dormitories (eight pumps in total). The type of sampling head was ATPS-20H, and the pump was MP-Σ3. All above instruments were manufactured by Sibata Scientific Technology Ltd. In each dormitory, one mini pump was used to collect fine particulate matter with glass fiber filter in the sampling head. The other pump collected PM_2.5_ with silicon fiber filter in the sampling head for analysis of carbonaceous constituents to determine the concentrations. All pumps continuously ran in 1.5 L/min constant flow to collect the particulate matter originated from indoor and outdoor environment in successive 24 hours. Glass and silicon fiber filters were replaced on 7:30 a.m. on every measurement day. Measurement from 7:30 a.m. one day to 7:30 a.m. the next day was considered a measurement day. Hobo temperature and humidity meters were also located at the selected dormitories indoors and outdoors to collect real-time temperature and humidity during sampling period. All glass and silicon fiber filters were placed in an atmosphere where temperature was 23 ± 0.2 °C and relative humidity was 50 ± 1% for 24 hours. Then, they were weighted twice by Microbalance (UMX-2, Mettler-Toledo, Inc., Columbus, OH). If the difference value was above 1 μg, a third weighing was performed and average two values that difference value was below 1 μg. Apart from this, 1 filter in every 20 filters was used as a blank control.

The mass concentrations of OC and EC in PM_2.5_ were measured by a thermal-optical transmittance analyzer (DRI Model 2001 Carbon Analyzer, Desert Research Institute, Las Vegas, NV). The sample was stepwise heated at 120 °C, 250 °C, 450 °C, and 550 °C in pure helium atmosphere to thermalize OC, and then at 550°C, 700°C, and 800°C to oxidize EC in 2% oxygen-contained helium atmosphere. Pyrolysis error was calibrated according to the reflectivity of filter paper.

Both carbonaceous fractions including primary organic carbon (POC) and secondary organic carbon (SOC) were estimated according to the equation [26]

(1) $${\mathrm{OC}}_{\sec}{=\mathrm{OC}}_{\mathrm{total}}-{\left(\frac{\mathrm{OC}}{\mathrm{EC}}\right)}_{min}\times \mathrm{EC}$$

${\mathrm{OC}}_{\sec}$ was SOC, ${\mathrm{OC}}_{\mathrm{total}}$ the total measured organic carbon, and ${\left(\frac{\mathrm{OC}}{\mathrm{EC}}\right)}_{min}$ the minimum value of OC/EC ratios. POC concentration was calculated by subtracting SOC concentration from total OC concentrations. This calculation was successfully applied to several previous studies estimating the concentrations of POC and SOC in cities of China [27,28]. 

#### 2.2.3. Statistical Analysis

The mixed-effect model was adopted in this study to analyze repeated measured data, with a random intercept for each subject. In single-pollutant model, considering that PEF and FEV_1_ data of subjects may fluctuate around their diverse mean values and the cumulativeness of PEF and FEV_1_ data of subjects in one day, we set the participant and day of measurement as a random effect. To control the effect of day-of-week in lung function data, the single-pollutant model included a day-of-week dummy variable; to control for different physical status of participants, the model included quantitative variables such as age and height. Meanwhile, the influence of temperature and relative humidity was controlled for natural cubic spline with 3 degrees of freedom. Namely, a separate model was performed for each of the components, adjusting for covariates including age, gender, height, day of week, day of season, season, temperature, and relative humidity. Except for age and height, other categorical covariates were set as dummy variables to be controlled. Because of the correlation between constituent and PM_2.5_, we then performed constituent-PM_2.5_ model to investigate the relationship between carbonaceous components in PM_2.5_ and effects independent of PM_2.5_. In order to explore the lag effect and cumulative effect of carbonaceous carbon of PM_2.5_ on physical health, we examined the models using multiple periods preceding lung function measurement. We fit the model using single lag 0 day, 3 day, and moving lags of 0–3 days. Lag 0 and lag 3 reflected the association of lung function change and air pollution 0 and 3 days before respectively. Lag 0–3 day was the average concentration of the periods and had cumulative health effects. To control the total mass of PM_2.5_, constituent-PM_2.5_ model was performed based on single-pollutant model. Separate models were further constructed for each constituent with PM_2.5_ mass. 

Models described above were conducted in R version 3.4.1 with *lme*4 package. All statistical tests were two-sided and *P* <0.05 was considered as significant.

## 3. Results

### 3.1. Descriptive Statistics of Participants, Pollutants, and Meteorology Variables

All 37 students completed the repeated measurements. Basic characteristics of participants enrolled in this panel study were shown in Table 1. Average height, weight and BMI of all subjects were 164.95cm, 54.7kg, and 20.08 kg/m^2^ respectively. Average height, weight, and BMI of male group and female group were 171.67cm, 59.1kg, 20.03 kg/m^2^ and 158.58cm, 50.6kg, 20.13 kg/m^2^ respectively. 

We summarized descriptive statistics of lung function measurements, concentrations of PM_2.5_, carbonaceous constituents and meteorology over nearly one-year follow-up in Table 2. 3328 person-times valid FEV_1_ (L/s) and 3538 person-times valid PEF (L/min) were collected totally, and patterns were similar in morning and evening. Generally, concentrations of outdoor pollutants were slightly higher than one of indoors, except for POC which showed higher level indoors. Average concentrations of indoor and outdoor PM_2.5_ were 80.47 μg/m^3^ and 96.77 μg/m^3^, respectively. PM_2.5_ concentrations during the study period exceeded WHO PM_2.5_ Guidelines (10 μg/m^3^ annual mean). Average indoor temperature was moderately higher than outdoor ones, while relative humidity was higher outdoors. Seasonal variations were also observed. Pollution was the most serious in winter, then sequentially in autumn, spring, and summer. Average PM_2.5_ concentrations in winter were 114.74 μg/m^3^, 148.76 μg/m^3^ indoors and outdoors, respectively, which are two times higher than 50.26 μg/m^3^ and 54.45 μg/m^3^ in summer (Table S1). Air pollution was similar in dormitories, since they were so close and in the same building.

### 3.2. Correlation Matrix between Indoor/Outdoor Pollutants 

Spearman correlation coefficients among indoor/outdoor pollutants were mostly statistically significant, indicating strong correlations among pollutants. Indoor PM_2.5_ concentration was closely correlated with outdoor PM_2.5_ concentrations (*r* = 0.94). Concentrations of indoor carbonaceous components were positively correlated with each other, with correlation coefficients ranging from 0.56 to 0.93. Spearman correlation coefficients of outdoor carbonaceous concentrations ranged from 0.81 to 0.98, indicating strong correlations of outdoor pollution. Correlations among indoor and outdoor concentrations of pollutants ranged from 0.65 to 0.95. Temperature was consistently negatively related with all pollutants, and relative humidity was weakly correlated with pollutants. (Table S2)

### 3.3. Estimated Effects of Carbonaceous Components on Lung Function

#### 3.3.1. Global Analysis of Health Effects of Carbonaceous Components

**Figure 1:**
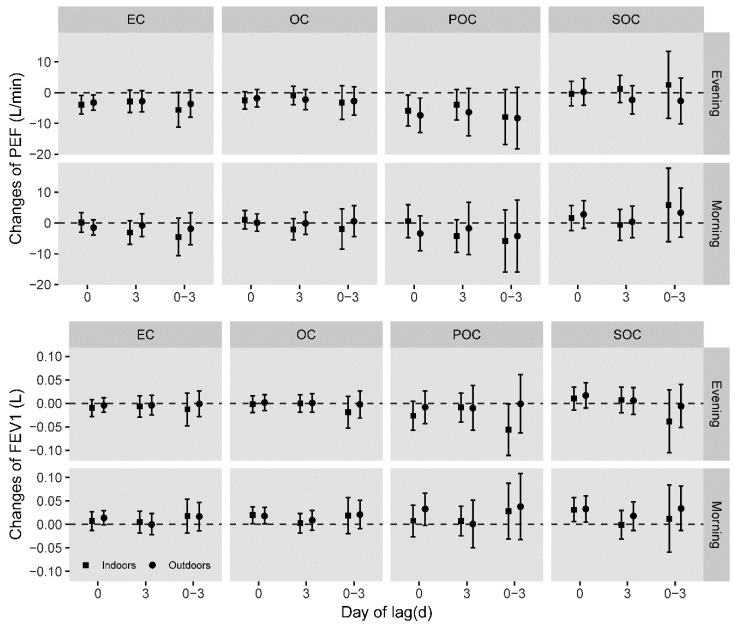
Estimated changes of morning/evening PEF and FEV_1_ associated with 10 μg/m^3^ increase of carbonaceous compositions in single-constituent model adjusting for gender, age, height, and season when the measurement was conducted, day of week, day of season, and meteorology. Squares indicate the effects of indoor compositions while circles indicate that of outdoor compositions.

The estimated effects of carbonaceous components on respiratory functions in single-constituent model were presented in Figure 1, with controlling gender, age, height, season factors during the conducted measurement, day of week, day of season, and meteorology for adjusting for potential confounders. Generally, patterns of health effects indoors and outdoors were alike. Indoor and outdoor EC concentrations were inversely associated with evening PEF at day0. A 10 μg/m^3^ increment of EC was significantly associated with −3.93 (95%*CI*: −6.89, −0.97) L/min and −3.21 (95%*CI*: −5.67, −0.75) L/min change in evening PEF indoors and outdoors, respectively. Indoor POC was negatively related with evening PEF at day0 and evening FEV_1_ at lag0-3 day, of which the estimated change was −5.82 (95%*CI*: −10.82, −0.81) L/min and −0.06 (95%*CI*: −0.11, 0.00) L, respectively. A 10 μg/m^3^ increment of outdoor POC concentration was significantly associated with −7.32 (95%*CI*: −12.93, −1.71) L/min change of evening PEF at day0. Unexpected positive associations with morning FEV_1_ were observed in OC and SOC in single-constituent model.

**Figure 2:**
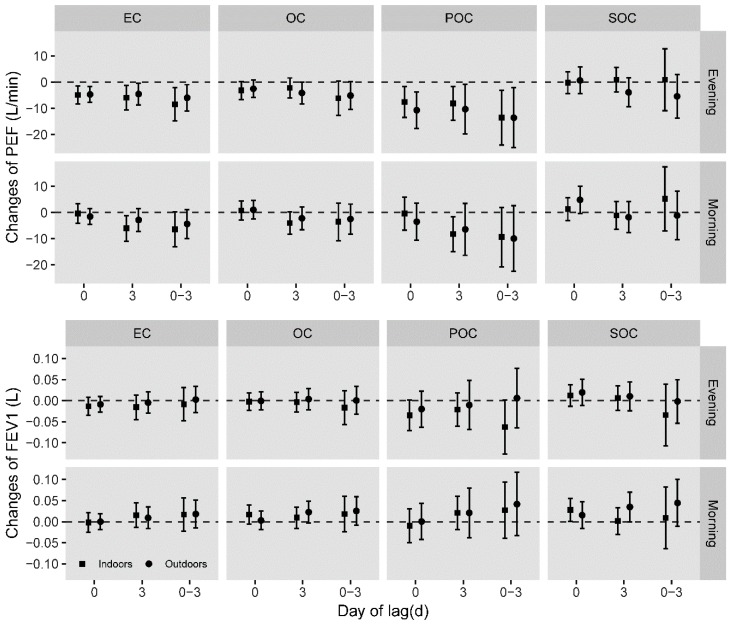
Estimated changes of morning/evening PEF and FEV_1_ associated with 10 μg/m^3^ increase of carbonaceous compositions indoors in constituent-PM_2.5_ model adjusting for total PM_2.5_ mass, gender, age, height, season when the measurement was conducted, day of week, day of season, and meteorology. Squares indicate the effects of indoor compositions while circles indicate that of outdoor compositions.

In constituent-PM_2.5_ model further controlling total mass of PM_2.5_, significant inverse associations between pollution and pulmonary functions were obvious in the evening. Negative relations between indoor EC concentrations and evening PEF were found in lag3 day and lag0-3 day. A 10 μg/m^3^ increment of indoor EC concentration was significantly associated with −5.95 (95%*CI*: −10.66, −1.23) L/min and −8.47 (95%*CI*: −14.83, −2.11) L/min changes of PEF at lag3 and lag0-3 day. Outdoor EC and POC concentrations were separately associated with decrements of evening PEF at day3 or lag0-3 day. Yet, positive relations were unexpectedly observed between SOC and morning FEV_1_ at day0 indoors (Figure 2). 

Conclusively, health effects of carbonaceous constituents on PEF were much stronger in the evening. Negative associations were mostly observed between EC and POC and evening PEF in both indoor and outdoor contexts. 

#### 3.3.2. Seasonal Analysis of Health Effects of Carbonaceous Components

To clarify if there was seasonal difference, a seasonal stratified analysis was conducted. Because of diverse concentrations of PM_2.5_ in seasons (Table S1), constituent-PM_2.5_ model was performed with controlling total PM_2.5_ mass, gender, age, height, day of week, day of season, and meteorology. The stratified analysis based on seasons indicated that participants’ evening PEF was the most sensitive to indoor and outdoor carbon fractions in winter. Negative associations between evening PEF and carbonaceous compositions were not found in spring or autumn. Most of the inverse health effects of carbons on evening PEF were observed in winter especially at lag0-3 day, indicating that carbonaceous fractions had cumulative effects on evening PEF (Table 3, Table 4). In indoor and outdoor environments, inverse health effects were stronger in winter than in overall seasons. For instance, 10 μg/m^3^ increases of indoor OC concentrations were not significantly related with −3.15 (−6.59, −0.30) L/min changes of evening PEF at lag0 day, but in winter, the pulmonary changes were −16.78 (−32.16, −1.40) L/min changes (Table 3). Comparative results of health effects between indoor and outdoor were of interest. Indoor overall health effects were less severe than outdoors. However, health effects of carbonaceous constituents were stronger indoors specifically in winter.

## 4. Discussions

This panel study examined the associations between carbonaceous components in PM_2.5_ and pulmonary function in a group of healthy university students in Wuhan, China. Estimated effects on impaired pulmonary function may be different in indoor and outdoor environment. However, currently, most of previous studies focused on outdoor pollution. Few studies investigated health effects of pollution in both indoor and outdoor contexts. Current studies regarding indoor/outdoor relationships about carbonaceous constituents in PM_2.5_ usually explored the characteristics of pollution [17,20,29] rather than its adverse health effects on human beings. Smoking, cooking, cleaning solvents, waxes, and cleaners/polishers could be major sources of indoor OC [30]. Unlike student’s studios and apartments in Europe, United States, and other countries and regions, the dormitories in mainland China are not equipped with kitchens. Without cooking and smoking, there were no obvious indoor carbonaceous pollutant sources of PM_2.5_, so penetration from outdoor pollutants might be the main reason for indoor pollutants, which could be also referred to strong correlations among indoor and outdoor constituents (Table S2). To our knowledge, this is the first study exploring associations between carbon compositions and lung function in both indoor and outdoor contexts in healthy young adults. This study found that carbonaceous fractions in PM_2.5_, measured both indoors and outdoors, were associated with adverse effects on respiratory function.

The inconsistency existed among previous panel studies regarding to the associations between carbonaceous compositions and respiratory function [11,14,18,28,31], probably due to diverse study designs, locations, participants with different features, varying air pollution characteristics and different indicators of impaired lung function. In the studies conducted among healthy adults, the inverse associations between carbonaceous components and pulmonary function did not show a consistent significance, even null in specific moving average days [28,31]. OC and POC were positively associated with forced vital capacity (FVC) at 3-day moving average [31], inversely associated with morning FEV_1_ at 7-day moving average [28], while there was consistent null associations between SOC and FEV_1_/PEF [28]. Ambient pollution might increase the risks and exacerbate existing vulnerabilities in specific populations with comorbidities, presenting greater impacts on their health. In an elderly cohort, SOC in PM_2.5_ was significantly positively associated with fractional exhaled nitric oxide (FE_NO_) which was an indicator of airway inflammation [14]. In elders with asthma, indoor/outdoor/personal BC (black carbon, similar with but not identical to EC) was also significantly associated with increases in FE_NO_ [18]. Carbonaceous contents, particularly EC, have posed adverse effects on lung function in COPD patients [11]. Yet unexpectedly, it was documented that in children with asthma being prescribed with bronchodilators, there was no significant association between FEV_1_ and particulate OC or EC [16]. In aging population with asthma or COPD, indoor/outdoor/personal BC did not significantly relate to spirometry measurements [18]. Among susceptible individuals in previous studies described above, effects of carbonaceous components were varying. Furthermore, few studies were conducted in healthy adults. Thus, we aimed to investigate associations between indoor/outdoor pollution and pulmonary function in healthy adults.

EC consistently presented robust negative effects on spirometry measurements, especially in evening PEF in indoor environment. After controlling the total PM_2.5_ mass, EC remained significantly inversely associated with evening PEF and FEV_1_ at lag3 day and moving average period. Results of EC in this study was consistent with a study in Shanghai, a megacity in which EC had robust detrimental effects on PEF or FEV_1_, and the effects were stronger than OC [11]. In adults with asthma, EC significantly decreased FEF_25-75_ while continuously increased FE_NO_ [12]. Although EC and OC were highly correlated, significant adverse effects on PEF and FEV_1_ were found in EC but null in OC [32]. Carbon core of EC particles is highly adsorptive [33], potentially adsorbing toxic substance which would go deeply into lung. In this way, EC would be a component of utmost significance to lead into impaired pulmonary function. Evidenced in previous publication, speed limit reduction from 120 km/h to 90 km/h in selected highway during winter leaded to great reduction of EC [34], so it might be a strategy to issue certain regulations to mitigate adverse health effects caused by EC.

In a single-constituent model, POC was negatively associated with evening PEF at day0. After controlling concentration of PM_2.5_, it remained significant with evening PEF outdoors at day3. A significant inverse association between SOC and respiratory measurement was found in this study. In patients with coronary artery disease, compared with SOC, POC triggered more increments of IL-6 that is an indicator of inflammation [35]. Also, it was documented that POC had larger effects on lung function than OC or SOC in healthy young adults in Beijing [31]. POC was a representative indicator of PM organics from combustion sources emission, which may lead to health effects reflecting the impact of traffic-related pollution [35,36]. The underlying mechanism may be as follows: polycyclic aromatic hydrocarbons bound to PM deposited in airway and stimulated oxidative reactions [14].

PEF was more sensitive than FEV_1_ in this study, particularly in the evening. A study of brief exposure to traffic pollution in a road tunnel failed to find any effect on FEV_1_ [28]. Likewise, another study found larger decrements in FEF_25-75_ than in FVC or FEV_1_ in a panel of asthma patients who exposed to traffic-related pollution [12]. Within a short-term period, pulmonary expiration rate rather than volume may be more sensitive to air pollution [31], suggesting that expiratory rate would be more proper to estimate response to pollution exposure than pulmonary volume [31]. In this study, evening respiratory measurements were more sensitive to pollution, partly due to circadian rhythm that could affect pulmonary function [37]. Another possible explanation was that the cumulative harmful effects of pollution on health manifested in evening. 

Positive associations between morning FEV_1_ and PM_2.5_ constituents were found in this study, which conflicted with anticipations that deleterious pollution would lead to adverse effects on lung function. A previous study, conducted among healthy university students in Beijing, also observed increased lung function after exposure to deteriorated pollution in short-term moving average [31]. In cyclists, the associations between air pollution and lung function during or immediately after riding a bicycle were mostly positive, while it would become negative 6 hours later, but it was not statistically significant [38], probably because of the modification of cycling intensity on physiology response to diesel exhaust exposure [39]. These findings were different from studies performed in vulnerable participants [11,12,16], possibly because of the variety of age, fitness, and physical activities. There was a breakeven point between the risk increments due to air pollution and the risk reduction due to physical activities [40]. Within the point, the risk from air pollution would be compensated by the health benefits from physical activities [40]. Healthy and young participants in this study were likely to have workout each day, so they might have better physical health and could mitigate adverse effects of ambient pollution. It was demonstrated that health risks were linearly increased with increased exposure to low-to-moderate air pollution, while the health benefits were curvy-linearly associated with increased level of physical activities [41]. In future study, variables about participants’ daily activities would be of significance to be controlled.

The adverse health effects were mostly observed in winter in this study (Table 3, Table 4). A multicity study in China also found largest health effect in winter, because coal has been the major energy source in China, particularly in winter for heating from coal-fired boilers and power plants [42]. In another nation-wide study, seasonal variations of PM on mortality were observed and largest health effects were found in winter and summer [42]. Specifically, in Wuhan, the strongest health effects of pollution for all-natural, cardiovascular, stroke, respiratory, and cardiopulmonary mortality occurred in winter, possibly caused by the highest concentration of particulate matter and gaseous pollutants and lowest temperature [43]. Daily lowest temperatures in Wuhan were often below 0 °C and the cold weather lasted for several weeks. The estimated strongest effects may be an indicator of the source-related component of PM [43], and PM composition data across seasons in this study evidenced the hypothesis. In Wuhan, concentrations of PM, OC, and EC were highest in winter [44,45]. This study also found highest concentrations of EC and POC and strongest health effects in winter. Thus, a relevant pollution control strategy should be enhanced in winter.

Several strengths of our study need to be stated here. Multiple repeated measurements on the same panel allowed us to control individual heterogeneity. Measurements were conducted in a set of four seasons, providing us more information about seasonal variance and diverse associations between pulmonary function and pollution. Concentrations of indoor pollutants were also obtained, instead of analyzing outdoor pollution-related health effects alone. Nevertheless, limitations are supposed to be addressed. Non-differential measurement errors were inevitable in this panel study due to the surrogate exposure of fixed-site monitoring, but all participants lived in the same campus and exposures might be similar. Determined by previous publications [39,40,46,47], benefits from activities like cycling and walking could outweigh the risks of ambient pollution. Yet physical activities and their intensity, as well as socioeconomic status, were not obtained and controlled in this study. 

## 5. Conclusions 

Carbonaceous components in PM_2.5_ indeed had impacts on pulmonary function, even in healthy young adults especially in evening and on PEF. EC and POC present more robust effects, so the government’s mitigation strategies are supposed to focus on reducing related pollution—diesel exhaust, combustion, and biomass burning.

## Figures and Tables

**Table 1:** Basic characteristics of participants.

Characteristics	All Subjects (*n* = 37)	Male (*n* = 18)	Female (*n* = 19)
Age, years	20.70 ± 0.80	21.00 ± 0.90	20.50 ± 0.60
Height, cm	164.95 ± 8.16	171.67 ± 5.87	158.58 ± 3.67
Weight, kg	54.7 ± 7.0	59.1 ± 5.62	50.6 ± 5.61
Body mass index, kg/m^2^	20.08 ± 1.84	20.03 ± 1.51	20.13 ± 2.1

**Table 2:** Descriptions of pulmonary function, pollutants, and meteorological variables.

	N	Mean	SD	Min	P_25_	Median	P_75_	Max
Pulmonary function								
Morning FEV_1_ (L)	1646	2.99	0.78	1.22	2.44	2.85	3.49	5.14
Evening FEV_1_ (L)	1682	2.99	0.77	1.01	2.44	2.84	3.42	5.24
Morning PEF (L/min)	1758	474.70	128.13	226.00	367.00	447.00	561.00	894.00
Evening PEF (L/min)	1780	476.70	125.15	250.00	374.00	445.00	562.00	838.00
Pollutant & meteorology								
Indoor								
PM_2.5_ (μg/m^3^)	54	80.47	39.93	23.07	48.46	65.40	107.52	189.42
OC (μg/m^3^)	54	14.11	10.24	1.35	7.25	9.75	18.30	42.43
EC (μg/m^3^)	54	10.88	8.65	1.12	5.81	8.05	12.98	55.37
SOC (μg/m^3^)	54	5.92	5.86	0.48	2.34	3.77	7.27	32.73
POC (μg/m^3^)	54	7.97	6.34	0.82	4.26	5.90	9.51	40.58
Temperature (°C)	54	20.85	8.31	9.18	13.38	18.43	29.99	37.18
Humidity (%)	54	61.45	9.20	36.28	55.71	62.80	67.60	77.89
Outdoor								
PM_2.5_ (μg/m^3^)	54	96.77	49.10	29.06	59.21	80.48	125.67	228.91
OC (μg/m^3^)	54	14.74	11.55	1.34	6.70	10.42	20.56	52.01
EC (μg/m^3^)	54	12.54	10.79	1.10	6.11	8.78	15.40	69.00
SOC (μg/m^3^)	54	8.92	7.73	0.58	3.63	5.79	12.87	33.27
POC (μg/m^3^)	54	5.50	4.73	0.48	2.68	3.85	6.75	30.25
Temperature (°C)	54	18.90	9.55	4.97	10.47	16.37	29.66	36.13
Humidity (%)	54	62.27	10.71	39.63	55.32	63.28	70.31	83.76

**Table 3:** Estimated health effects on evening PEF (L/min) with 10 μg/m^3^ increase of indoor pollution in constituent-PM_2.5_ model in different periods.

Components	Lag	β (95%*CI*)				
		Overall	Spring	Summer	Autumn	Winter
OC						
	0	−3.15 (−6.59, 0.30)	−20.43 (−68.89, 28.04)	−18.77 (−76.7, 39.17)	−1.25 (−16.47, 13.97)	−16.78 (−32.16, −1.40) *
	3	−2.21 (−6.00, 1.58)	−2.21 (−188.26, 119.55)	4.54(−54.67, 63.75)	−10.46 (−31.69, 10.78)	−16.36 (−96.91, 64.20)
	0-3	−6.14 (−12.63, 0.35)	−37.62 (−245.38, 170.14)	−48.39 (−184.19, 87.42)	15.45 (−61.85, 92.75)	−98.02 (−216.65, −20.60) *
EC						
	0	−4.88 (−8.34, −1.42) *	−23.08 (−78.72, 32.55)	−93.39 (−166.93, −19.85) *	−6.41 (−15.63, 2.81)	−3.08 (−12.14, 5.97)
	3	−5.95 (−10.66, −1.23) *	−156.25 (−527.36, 214.86)	−13.22 (−90.22, 63.79)	−2.88 (−11.27, 5.50)	16.69 (−34.68, 68.05)
	0-3	−8.47 (−14.83, −2.11) *	−160.30 (−679.83, 359.23)	−87.54 (−255.52, 80.44)	−24.26 (−60.82, 12.30)	−53.01 (−250.37, 144.34)
SOC						
	0	−0.19 (−4.36, 3.98)	−43.15 (−159.15, 72.84)	36.67 (−36.00, 109.33)	5.86 (−4.99, 16.71)	−18.95 (−50.54, 12.65)
	3	0.90 (−3.78, 5.58)	−23.05 (−215.99, 169.90)	14.63 (−68.14, 97.40)	1.76 (−14.21, 17.73)	−13.93 (−58.04, 30.18)
	0-3	0.94 (−10.91, 12.80)	−27.74 (−318.44, 262.97)	−29.08 (−316.27, 258.10)	44.52 (−8.25, 97.28)	−112.76 (−257.20, 31.68)
POC						
	0	−7.56 (−13.43, −1.69) *	−31.51 (−107.45, 44.43)	−127.43 (−227.77, −27.09) *	−8.75 (−21.33, 3.83)	−13.51 (−29.50, 2.48)
	3	−8.11 (−14.55, −1.67) *	−213.20 (−719.60, 293.19)	−18.06 (−123.30, 87.19)	−3.94 (−15.38, 7.51)	22.77 (−47.31, 92.85)
	0-3	−13.56 (−24.02, −3.10) *	−218.73 (−927.63, 490.17)	−119.40 (−348.35, 109.55)	−33.11 (−82.99, 16.78)	−53.85 (−136.23, 28.53)

*P < 0.05.

**Table 4:** Estimated health effects on evening PEF (L/min) with 10 μg/m^3^ increase of outdoor pollution in constituent-PM_2.5_ model in different periods.

Components	Lag	β (95%*CI*)				
		Overall	Spring	Summer	Autumn	Winter
OC						
	0	−2.50 (−5.89, 0.90)	16.42 (−34.70, 67.55)	−53.46 (−115.36, 8.43)	−13.12 (−87.38, 61.15)	−9.38 (−22.33, 3.57)
	3	−4.11 (−8.24, 0.02)	−0.55 (−57.01, 55.91)	0.89 (−39.08, 40.85)	4.02 (−30.04, 38.07)	−15.17 (−29.65, −0.69) *
	0-3	−5.09 (−10.42, 0.23)	150.43 (−18.46, 319.32)	177.62 (−28.14, 383.38)	−31.56 (−123.76, 60.65)	−35.86 (−69.12, −2.61) *
EC						
	0	−4.69 (−7.72, 1.67) *	35.18 (−32.10, 102.47)	−57.12 (−130.31, 16.08)	1.81 (−12.74, 16.37)	−4.96 (−10.74, 1.57)
	3	−4.51 (−8.64, −0.38) *	−74.06 (−148.49, 0.36)	15.22 (−40.73, 71.18)	13.62 (−22.73, 49.97)	−9.78 (−19.63, 0.07)
	0-3	−5.95 (−10.98, −0.93) *	−10.42 (−172.92, 151.07)	85.09 (−72.04, 242.22)	−13.00 (−83.35, 57.35)	−28.18 (−60.02, 3.65)
SOC						
	0	0.67 (−4.44, 5.77)	10.90 (−50.61, 72.41)	−67.75 (−153.26, 17.76)	−3.89 (−29.05, 21.28)	−4.82 (−19.36, 9.71)
	3	−3.87 (−9.43, 1.70)	26.81 (−42.23, 95.85)	−5.08 (−62.29, 52.14)	−2.56 (−51.92, 46.80)	−17.65 (−42.80, 7.5)
	0-3	−5.40 (−13.78, 3.00)	285.81 (58.50, 513.13) *	396.14 (−12.91, 805.20)	−87.18 (−271.56, 97.20)	−24.01 (−58.69, 10.67)
POC						
	0	−10.69 (−17.60, −3.79) *	80.19 (−73.29, 233.67)	−135.14 (−307.95, 37.68)	4.14 (−29.05, 37.33)	−10.47 (−24.51, 3.58)
	3	−10.29 (−19.71, −0.87) *	−168.94 (−338.70, 0.81)	34.63 (−92.88, 162.15)	31.07 (−51.85, 113.99)	−22.31 (−44.78, 0.15)
	0-3	−13.58 (−25.04, −2.11) *	−23.66 (−393.03, 344.70)	193.58 (−159.19, 546.36)	−29.66 (−190.14, 130.83)	−64.29 (−136.91, 8.32)

*P < 0.05.

## Supplementary Materials

The following are available online at https://www.mdpi.com/1660-4601/16/13/2259/s1, Table S1: Average concentrations of pollutants and meteorology variables in each season (μg/m3). Table S2: Spearman correlation coefficients matrix among indoor and outdoor pollutants and meteorology variables.
