# Supplementary material for: Short-Term Effects of Carbonaceous Components in PM2.5 on Pulmonary Function: A Panel Study of 37 Chinese Healthy Adults
Source: Int J Environ Res Public Health. 2019 Jun 26;16(13):2259. doi: 10.3390/ijerph16132259 (PMC6651261; doi:10.3390/ijerph16132259)
Supplement: Supplementary file 1 [file ijerph-16-02259-s001.pdf]

Table S1. Average concentrations of pollutants and meteorology variables in each season (μg/m³)

	Spring	Summer	Autumn	Winter
Indoor				
$PM_{2.5}$	65.09±16.89	50.26±16.05	95.28±42.26	114.74±40.30
OC	8.47±2.14	6.32±2.94	21.54±11.92	21.15±8.65
EC	6.75±2.03	5.54±2.88	14.42±7.91	17.53±11.37
SOC	3.52±1.15	2.26±1.08	10.98±8.55	8.63±3.56
POC	4.95±1.49	4.06±2.11	10.57±5.79	11.77±6.41
Temperature (°C)	18.10±2.24	33.46±2.11	20.35±2.98	11.47±1.09
Humidity (%)	57.69±6.18	67.37±7.40	57.27±11.49	63.32±6.80
Outdoor				
$PM_{2.5}$	79.50±16.66	54.45±17.40	108.96±46.23	148.76±46.43
OC	8.23±2.28	6.12±3.05	22.45±12.48	23.33±11.14
EC	7.72±2.13	5.59±3.11	15.65±8.68	22.11±14.67
SOC	4.84±1.59	3.67±1.79	15.59±9.38	14.20±5.53
POC	3.39±0.93	2.45±1.36	6.86±3.81	9.69±6.43
Temperature (°C)	16.02±3.33	33.16±1.59	18.11±4.45	8.30±2.05
Humidity (%)	61.53±11.14	67.13±5.47	63.88±12.95	56.59±8.96

Table S2. Spearman correlation coefficients matrix among indoor and outdoor pollutants and meteorology variables

	Indoor							Outdoor						
	$PM_{2.5}$	OC	EC	POC	SOC	Tempt (°C)	RH [a] (%)	$PM_{2.5}$	OC	EC	POC	SOC	Tempt (°C)	RH [a] (%)
Indoor														
$PM_{2.5}$ (μg/m³)	1.00													
OC (μg/m³)	0.79*	1.00												
EC (μg/m³)	0.86*	0.93*	1.00											
POC (μg/m³)	0.85*	0.93*	1.00*	1.00										
SOC (μg/m³)	0.56*	0.87*	0.68*	0.68*	1.00									
Tempt (°C)	-0.44*	-0.46*	-0.40*	-0.38*	-0.47*	1.00								
RH [a] (%)	0.22*	0.12*	0.21*	0.20*	-0.01	0.21*	1.00							
Outdoor														
$PM_{2.5}$ (μg/m³)	0.94*	0.83*	0.85*	0.85*	0.65*	-0.57*	0.19*	1.00						
OC (μg/m³)	0.77*	0.91*	0.90*	0.89*	0.76*	-0.47*	0.14*	0.82*	1.00					
EC (μg/m³)	0.84*	0.91*	0.95*	0.95*	0.70*	-0.49*	0.18*	0.89*	0.94*	1.00				
POC (μg/m³)	0.84*	0.91*	0.95*	0.95*	0.70*	-0.49*	0.18*	0.89*	0.94*	1.00	1.00			
SOC (μg/m³)	0.76*	0.92*	0.87*	0.87*	0.79*	-0.47*	0.12*	0.81*	0.98*	0.90*	0.90*	1.00		
Tempt (°C)	-0.34*	-0.39*	-0.30*	-0.29*	-0.45*	0.92*	0.29*	-0.49*	-0.40*	-0.40*	-0.40*	-0.40*	1.00	
RH [a] (%)	0.08	-0.05	0.05	0.04	-0.12*	0.39*	0.63*	-0.01	-0.04	-0.02	-0.02	-0.04	0.29*	1.00

[a] RH, abbreviation of relative humidity; *P<0.001
